# Editorial: Clinical and Molecular Features of Infectious Cardiomyopathies: Implications for Prevention, Treatment and Management

**DOI:** 10.3389/fcvm.2022.952189

**Published:** 2022-06-10

**Authors:** Maria do Carmo Pereira Nunes, Lívia Silva Araújo Passos, Walderez Ornelas Dutra

**Affiliations:** ^1^Graduate Program in Infectology and Tropical Medicine, School of Medicine, Federal University of Minas Gerais, Belo Horizonte, Brazil; ^2^Brigham and Women's Hospital, Harvard Medical School, Boston, MA, United States; ^3^Cell-Cell Interactions Laboratory, Department of Morphology, Institute of Biological Sciences, Federal University of Minas Gerais, Belo Horizonte, Brazil; ^4^Instituto Nacional de Ciência e Tecnologia em Doenças Tropicais (INCT-DT), Salvador, Brazil

**Keywords:** cardiomyopathy, biomarkers, pathology, Chagas disease—*Trypanosoma cruzi*, infectious diseases

Heart diseases are the leading cause of death worldwide. The current Research Topic covers aspects associated with the pathogenesis or progression of different cardiomyopathies, ranging from the role of the pathogen, through the influence of diet and host factors. Parasitic infections, which are often associated with cardiac manifestations, account for significant morbidity and mortality in extensive areas of the world, especially in developing countries. Some of these infections may affect the anatomic structures of the heart, such as the myocardium and pericardium, as well as the pulmonary vasculature, producing a wide variety of cardiac manifestations. As an example, Chagas disease, caused by the protozoan *Trypanosoma cruzi*, is a major cause of cardiomyopathies worldwide.

Although the articles presented in the Research Topic discuss myocardial diseases of different etiologies, they are heavily biased toward Chagas disease cardiomyopathy, which is the main Research Topic of eight out of 10 papers. The study by de Oliveira et al. addressed an important issue in Chagas disease, which is the correlation of parasitemia and disease severity. Studying a cohort of 181 patients, they demonstrated that, although the parasitemia is variable amongst chronic patients, high parasitemia is correlated with a reduction of left ventricular ejection fraction, an important prognostic factor in Chagas cardiomyopathy. Intriguingly, the systematic review presented by Bosch-Nicolau et al. in this same Research Topic failed to demonstrate an association between parasite DNA and Chagas cardiomyopathy. Although apparently antagonistic, these studies consider different aspects: myocardial function in cardiac patients, and cardiomyopathy without considering severity of heart damage. These studies add to our knowledge in an area that is still controversial and need further development.

In another study, de Oliveira et al. demonstrated the occurrence of a moderate to high intra-discrete typing unit (DTU) V variability in patients from Bolivia living in Spain. Importantly, this study showed an association between parasite alleles with the lack of cardiac involvement in patients with Chagas disease, suggesting that parasite genetic intra-DTU variability may be a marker of disease favorable outcome. Echeverría et al. approached the potential use of several molecules as biomarkers of left ventricular diastolic dysfunction. The authors showed that, except for sST2, the levels of all markers analyzed [pro-B type natriuretic peptide (NT-proBNP), galectin-3 (Gal-3), neutrophil gelatinase-associated lipocalin (NGAL), high-sensitivity troponin T (hs-cTnT), soluble (sST2), and cystatin-C (Cys-c)] were associated with high risk of patients having diastolic dysfunction. Amongst these markers, NT-proBNP was the best marker for identifying diastolic dysfunction. These studies, although with relatively low numbers of patients, enlighten the search for much needed markers of disease severity.

The role of the host's immune response was also addressed in this Research Topic. Pinto et al. showed a modulation of activated and regulated T-cell subpopulations by the interactions of CD80/CD86 with CD28/CTLA-4, respectively. This work implicates the network of co-stimulatory signals as potential targets to control Chagas cardiomyopathy. The study by Neves et al. shows an extensive comparison of the expression of cytokines, recruitment and effector molecules between patients with Chagas and idiopathic cardiomyopathies. While the data shows significant immunological differences between the cardiomyopathies, some similarities regarding expression of chemokine receptors and TNF were found, identifying potential common targets of intervention in these diseases. Using experimental models of *T. cruzi* infection, de Souza et al. and Dhanyalayam et al. showed the involvement of a lard-rich diet and of sex in severity of Chagas cardiomyopathy, respectively. These findings contribute to our understanding regarding the role of host and environmental factors in Chagas cardiomyopathy involvement. This, the Research Topic of studies regarding Chagas disease in the current Research Topic, address the influence of parasite, host, and environmental factors in disease development.

Lastly, the studies by Ferrari et al. and Jan et al. discuss pulmonary arterial hypertension, a well-known consequence of infection with the parasite *Schistosoma mansoni*, and acute heart failure associated with Takotsubo syndrome, respectively. These studies highlight potential points of intervention and clinical markers of these diseases.

Overall, the studies presented here add to the current clinical knowledge of these cardiomyopathies, while also providing information regarding their epidemiology, pathogenesis, diagnosis, and treatment. In addition, the studies present strong suggestions of biomarkers of disease progression and/or cure, to guide patient care. We believe that the content of this Research Topic will aid general practitioners and cardiologists in the identification and treatment of parasitic and infectious cardiomyopathies. Figure 1 shows the graphic summary of the papers published in this topic.

**Figure 1:**
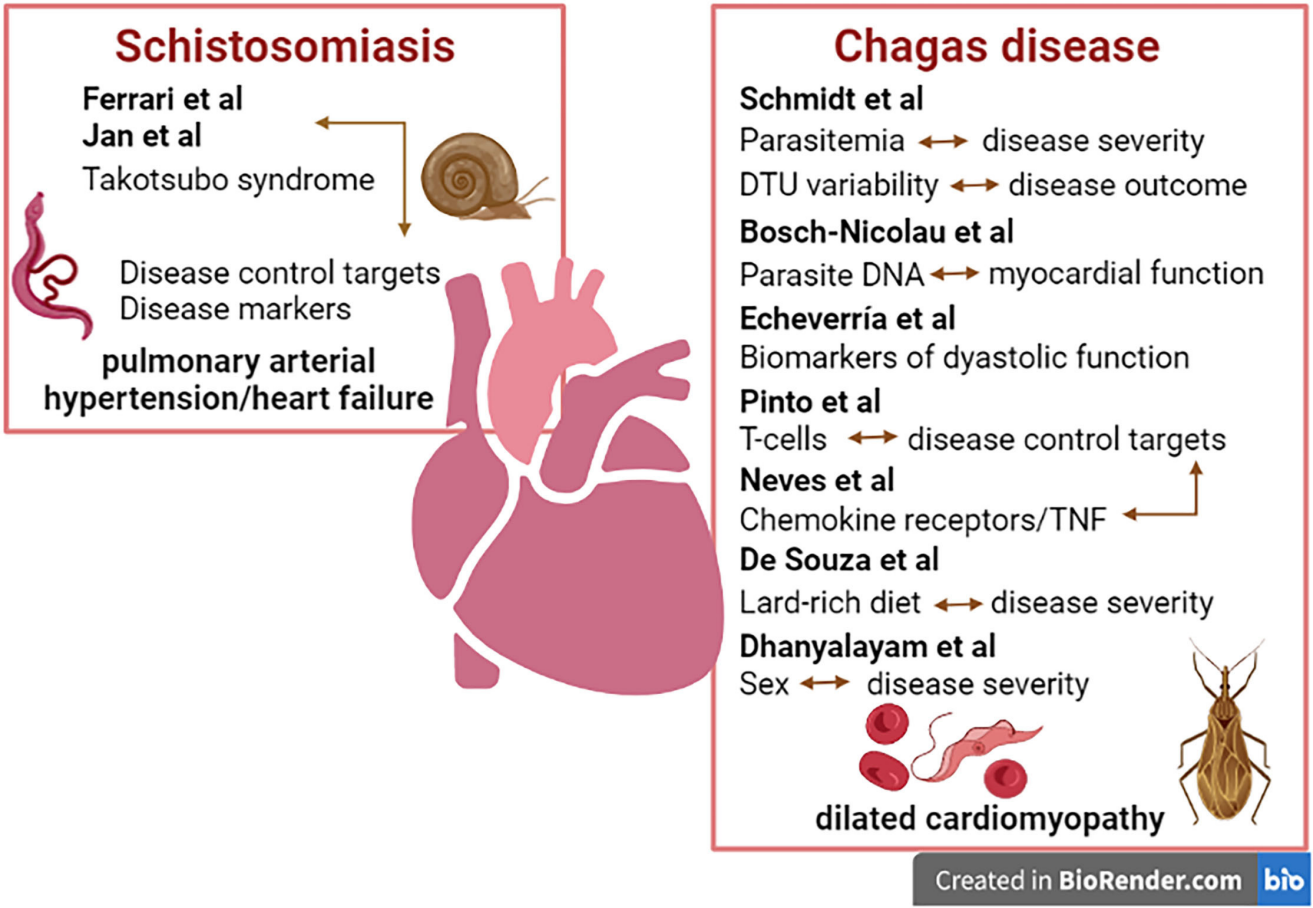
Graphic summary of the infectious cardiomyopathies addressed in this topic.

## Author Contributions

All authors listed have made a substantial, direct, and intellectual contribution to the work and approved it for publication.

## Funding

The authors are supported by CNPq, FAPEMIG, INCT-DT, and NIH R01AI138230 grants.

## Conflict of Interest

The authors declare that the research was conducted in the absence of any commercial or financial relationships that could be construed as a potential conflict of interest.

## Publisher's Note

All claims expressed in this article are solely those of the authors and do not necessarily represent those of their affiliated organizations, or those of the publisher, the editors and the reviewers. Any product that may be evaluated in this article, or claim that may be made by its manufacturer, is not guaranteed or endorsed by the publisher.

